# Resolution of anhedonia-like symptoms in patients treated for obesity with tirzepatide: A three-case series

**DOI:** 10.1016/j.obpill.2026.100302

**Published:** 2026-07-14

**Authors:** Spencer Nadolsky, Summer Kessel, Zachary A. Krumm, Grant M. Tinsley

**Affiliations:** a Vineyard Health, Holland, MI, USA; b Department of Neuroscience, University of Florida, Gainesville, FL, USA; c McKnight Brain Institute, University of Florida, Gainesville, FL, USA; d Department of Kinesiology & Sport Management, Texas Tech University, Lubbock, TX, USA

**Keywords:** Anhedonia, Case report, GLP-1, Obesity, Tirzepatide

## Abstract

**Introduction:**

Glucagon-like peptide-1 receptor agonists (GLP1RAs) have demonstrated substantial efficacy for obesity and metabolic disease treatment, with emerging evidence suggesting effects on reward-processing pathways and motivated behaviors. Although reductions in food craving and addictive behaviors may be beneficial, some patients anecdotally report diminished motivation and anhedonia-like symptoms during high-dose therapy. This case series highlights a potentially underrecognized neurobehavioral effect associated with high-dose tirzepatide and explores clinical management strategies.

**Case presentation:**

Three female patients with obesity treated with high-dose tirzepatide (15 mg·week^−1^) reported reduced motivation, emotional “flatness,” or loss of interest in exercise and previously enjoyable activities despite successful weight loss. Two patients had no prior history of mood disorders, while one patient had a history of depression and anxiety. Symptoms were described as distinct from depressive episodes and emerged after prolonged treatment at or near maximal dosing. One patient independently noted symptom improvement after temporary discontinuation and dose reduction surrounding a medical procedure.

**Interventions and outcomes:**

All patients underwent tirzepatide dose reduction to 10 mg·week^−1^ or lower. Two patients experienced marked improvement in motivation and enjoyment of daily activities after dose reduction alone. One patient required adjunctive bupropion therapy, which produced additional symptomatic improvement. Re-escalation of the tirzepatide dose in one patient resulted in recurrence of symptoms without additional weight loss benefit, whereas subsequent dose reduction restored motivation while maintaining weight-loss outcomes. Across all cases, symptom improvement occurred alongside continued weight maintenance or further weight reduction.

**Conclusion:**

This case series suggests that anhedonia-like symptoms may occur in a subset of patients receiving high-dose tirzepatide, potentially related to GLP1RA-mediated modulation of mesolimbic dopaminergic pathways. Clinicians should consider monitoring for changes in motivation and reward perception during treatment, particularly at higher doses. Dose reduction, with or without adjunctive dopaminergic therapy such as bupropion, may alleviate symptoms while preserving therapeutic benefits.

## Graphical abstract

**Figure:**
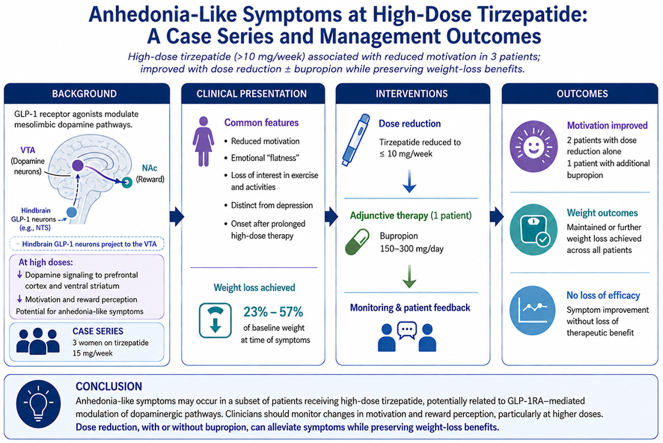

## 1 Introduction

Glucagon-like peptide-1 (GLP-1) receptor agonists have revolutionized the fields of obesity and metabolic medicine in the past 25 years, offering patients treatment efficacy for obesity and diabetic-related conditions that is matched only by metabolic/bariatric surgery [[1], [2], [3]]. GLP-1 receptor agonists (GLP1RAs) are thought to drive weight loss via their influence on feeding behaviors by simultaneously reducing basal hunger drive and by modulating the reward-perception of hyperpalatable foods [[4], [5], [6], [7], [8]]. Epidemiological and retrospective analysis has suggested that this drug class may be efficacious in treating a number of conditions beyond the well-established cardiometabolic indications, including many conditions involving reward-driven consumptive behaviors, neurocognitive disease, and rheumatologic conditions. Over 30 clinical trials investigating the efficacy of both GLP1 receptor mono-agonists (e.g., Semaglutide) and multi-agonists (e.g., Tirzepatide, Brenipatide) are now underway [9], with recent randomized clinical trial evidence demonstrating once-weekly Semaglutide injection (2.4 mg, s.c.) resulted in a 13.7% placebo-adjusted reduction in heavy drinking days with systemically positive changes in secondary outcomes [10]. Cumulatively, these results point to significant modulation of mesolimbic reward-processing circuitry by GLP1RAs, with significant impact on motivated behavior, value perception, reward prediction error, and cue-based responses [[11], [12], [13]].

**Fig. 1:**
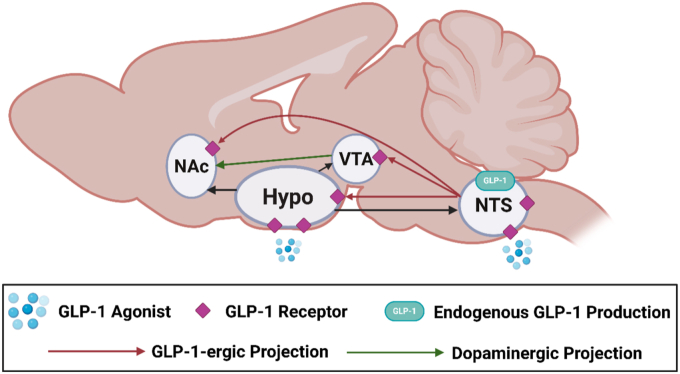
Relationships between GLP-1 receptor agonists and selected brain regions involved in responses to rewarding stimuli. *NAc: Nucleus Accumbens; VTA: Ventral Tegmental Area; NTS: Nucleus Tractus Solitarius; Hypo: hypothalamus*.

Reductions in “craving” for hyperpalatable and calorically dense foods as well as reduced compulsion to consume alcohol, nicotine, substances of abuse, or gambling, are all consistent with normalization of dopamine-driven “wanting” of cued high-value behaviors, and reports for less response to engagement with those activities are consistent with a reduced level of perceived “liking” of these otherwise highly rewarding behaviors. Preclinical evidence has demonstrated task-dependent alterations in mesolimbic dopamine responses where dopamine output in the Nucleus Accumbens (NAc) is suppressed in response to rewarding stimuli [14,15] (Fig. 1). However, this remains an active area of investigation, as other studies have found nominal or increased dopamine responses to valuable reward in the presence of GLP1RAs [16,17]. While the shifts in dopamine signaling may be beneficial in the context of reducing appetitive behavior in the context of addiction and overconsumption or rewarding/valuable substances, a potential consequence of mesolimbic and mesocortical dopamine signaling modulation is reducing basal or evoked dopamine release that is responsible for moment-to-moment motivational drive [18]. Indeed, neurodegenerative or chemical lesions of dopamine signaling in the dorsal striatum severely impair effortful movement [[19], [20], [21]], including in pursuit of food, despite no meaningful alteration in the liking of food once it is consumed [20]. Given these well-characterized phenomena, it is interesting that a small percentage of patients treated with GLP1RAs, especially the modern generation of the class at high doses, anecdotally report anhedonia-like symptoms and changes for motivation to engage in everyday activities [22]. It should be emphasized that this phenomena is distinct from symptoms of clinical depression, anxiety, and suicidality, where GLP1RAs have been generally been associated with non-significant or improved outcomes on average in well-controlled studies [[23], [24], [25], [26], [27], [28], [29]].

The role of GLP1RAs in anhedonia and changing self-perceived motivation for daily tasks has not been well characterized to date and warrants more direct consideration based on both patient reports and mechanistic feasibility. Therefore, this report presents a case series of three patients who reported anhedonia-like symptoms on high (>10 mg) doses of tirzepatide that have been treated via dose modulation and complementary dopamine-influencing medications. While considering efficacy versus side-effect profiles when choosing dosing strategies is always a patient-specific decision, this is intended to introduce anhedonia-like symptoms as a possible side effect, especially at high doses, and consider potential strategies for mitigating this side-effect while still preserving the profound efficacy of GLP1RA treatment.

## 2 Case Presentation

### 2.1 Patient information

Patient 1 was a 62-year-old female with a history of class 1 obesity (pre-treatment BMI 34.0 kg m^−2^) and hypertension and no prior history of depression or mood disorders. The patient had lost 26% of her weight while being treated with subcutaneous tirzepatide over the course of 19 months, up to a dose of 15 mg·week^−1^. The patient joined our online clinic to help optimize her body composition and overall health. Our clinicians introduced her to strength training and recommended dietary changes.

Patient 2 was a 36-year-old female with a history of class 1 obesity (pre-treatment BMI 33.9 kg m^−2^) along with depression and anxiety. The patient had lost 23% of her weight while being treated with 15 mg·week^−1^ of subcutaneous tirzepatide over the previous 24 months. She had previously used bupropion but had not taken it for several years. The patient joined our online clinic after seeing an online discussion of potential anhedonia precipitated by tirzepatide use.

Patient 3 was a 64-year-old female with a history of class 3 obesity (pre-treatment BMI 49.9 kg m^−2^) and no history of depression or mood disorders. The patient had lost 57% of her weight while being treated with 15 mg·week^−1^ of subcutaneous tirzepatide. The patient was not aware of subjective mood disturbances at this point.

### 2.2 Clinical findings

Patient 1 described feeling a lack of motivation to exercise and loss of interest in her other hobbies.

Patient 2 described feeling “flat” and unmotivated despite her weight loss success. She noted that these feelings differed from her feelings when previously diagnosed with depression. The patient also described being aware that she should exercise but being unable to “get herself to do it.”

Patient 3 briefly stopped tirzepatide due to a medical procedure.

### 2.3 Timeline

**Fig. 2:**
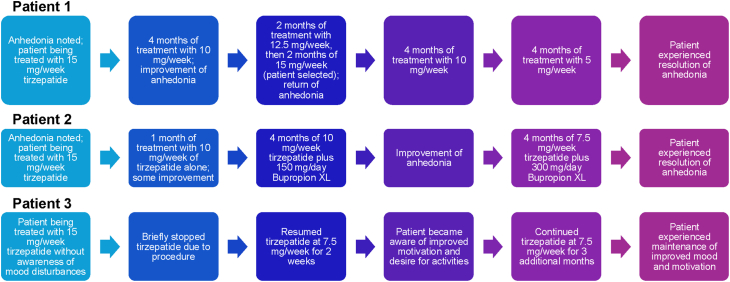
Patient timeline.

The timeline for each patient is displayed in Fig. 2.

### 2.4 Diagnostic assessment

Patient 1 was interviewed concerning past and current tirzepatide treatment, weight loss, and current lack of motivation and loss of interest in hobbies. Patient 2 was interviewed concerning past and current tirzepatide treatment, weight loss, history of depression, past bupropion use, and current feelings of “flatness.” Patient 3 was interviewed concerning past and current tirzepatide treatment, weight loss, and changes in medication use surrounding her medical procedure. In all cases, the presence of anhedonia was assessed through patient screening and clinical history taking. Anhedonia was established based on the loss of pleasure in previously rewarding experiences after ruling out organic or biological causes (e.g., hormonal, anemia, nutritional deficiencies, and sleep disturbances) and signs of depression other than anhedonia.

### 2.5 Therapeutic intervention

Patient 1 was prescribed a reduced dose of 10 mg·week^−1^ of subcutaneous tirzepatide for four months, during which the patient's desire to exercise and continue past hobbies improved markedly.

Patient 2 was prescribed a reduced dose of 10 mg·week^−1^ of subcutaneous tirzepatide for four weeks, during which she noticed significant improvements in her “flat” feelings but did not experience full resolution. At that point, 150 mg·day^−1^ bupropion XL was prescribed, which further improved her feelings within several weeks.

Patient 3 was required to temporarily stop her medication due to a procedure and resumed tirzepatide at a dose of 7.5 mg·week^−1^.

### 2.6 Follow-up and outcomes

Following the initial resolution of anhedonia, Patient 1 chose to self-administer a 12.5 mg·week^−1^ dose of subcutaneous tirzepatide for two months, followed by a 15 mg·week^−1^ dose for an additional two months, due to frustration with stalled weight loss. During this time, the patient's lack of motivation quickly returned, and no further weight loss was achieved. At this point, the patient was transitioned back to 10 mg·week^−1^ tirzepatide for four months, which resulted in improved motivation but some continued “flatness.” As such, the dose was further reduced to 5 mg·week^−1^ tirzepatide for four additional months. At this dose, the patient maintained previous weight loss while the lack of motivation and prior loss of interest in hobbies and daily activities completely resolved.

Patient 2 lost another 15 pounds (7% of original weight) during 4 months of treatment with 10 mg·week^−1^ tirzepatide and 150 mg bupropion. The patient reported feeling more motivated to participate in the activities she had stopped performing when being treated with 15 mg·week^−1^ tirzepatide without bupropion but desired further improvement. Bupropion was subsequently increased to 300 mg·day^−1^ while tirzepatide was decreased to 7.5 mg·week^−1^. During the subsequent four months, the patient lost an additional 15 pounds and reported feeling “her best.”

Within two weeks of reducing the dose of tirzepatide, Patient 3 experienced a notable improvement in her desire to complete daily activities she previously did not realize had been affected (i.e., retrospective recognition of reduced drive and motivation). Over the course of three months, she maintained the 7.5 mg·week^−1^ dose of tirzepatide. This resulted in weight maintenance but substantially improved motivation and desire to perform daily activities.

## 3 Discussion

Here we present a case series involving three individuals who reported changes in motivation to perform certain tasks consistent with anhedonia symptoms. These symptoms occurred on doses of tirzepatide that were at or near the maximum current dosing range (>10 mg), and notably the symptoms were distinct from depressive or anxiety-like symptoms. Symptom-directed lowering of the treatment dose of tirzepatide, with or without add-on bupropion for short- or long-term symptom management, largely alleviated these symptoms, and the patients were able to return to typical functioning. This phenomenon remains under-investigated in patients taking high-dose GLP1RAs despite anecdotal reports of anhedonia from some percentage of patients. Formal assessments and true frequency of these types of symptoms are complicated by inconsistency of treatment continuity, variable contact with clinical providers, and diversity in source of prescription or gray-market product use by patients.

Anhedonia is a mechanistically plausible impact of high-dose GLP1RA treatment based on its engagement and modulation of dopamine neuron cell bodies in the ventral tegmental area (VTA) that prominently influence moment-to-moment motivation for motor, cognitive, and complex executive behavior [14,[30], [31], [32]]. This may be described as a reduction in “wanting” of a previously appealing item or activity, as this pathway is intrinsically necessary for driving behaviors that drive pursuit of a reward or outcome [18,33]. Indeed, in preclinical trials, high-dose GLP1RA treatments have been demonstrated to reduce activity and alter pursuit of valuable rewards consistent with influencing midbrain dopamine signaling to the striatum, amygdala, and prefrontal cortex (PFC) [[34], [35], [36]]. Notably, these symptoms should not automatically be considered to be an onset of anhedonia in these animal studies, as these high dose treatments often reflect initial dose escalations in which patients experience nausea, fatigue, and symptoms related to engagement of neural circuits that are classically traced to the area postrema and nucleus tractus solitarius neurons that transmit responses to noxious stimuli [37,38]. However, emerging evidence of long-term dosing in both of animal and humans with this drug class does suggest that they alter pursuit of reward with task-dependent shifts in long-term behaviors [17,39,40]. The symptoms reported in this study are distinct from initial dose-escalation symptoms, as patients have acclimated to the initial dose escalation symptoms of these medicines while still reporting a level of anhedonia that is inconsistent with perceived GI or nausea symptoms. Therefore, we consider the possibility that modulation of the mesolimbic (VTA→NAc) and the mesocortical (VTA→PFC) dopaminergic circuits, which are necessary for motivation to pursue immediate and complex goals related to movement (with additional input from substantia nigra to dorsal striatum dopaminergic circuits), cognitive effort, reinforcement learning, and complex executive planning, show distinct output changes and potential suppression of dopamine output in patients treated with near-maximal doses of this drug class [[41], [42], [43], [44], [45], [46], [47]]. In particular, because it is known that significant reductions in VTA-derived dopaminergic output results in anhedonic-like states [[48], [49], [50]], both clinical vigilance and further research to study which patients may be at risk and how to mitigate symptoms is likely warranted.

## 4 Limitations

Several limitations of the current work should be noted. First, as a case study, the present observations are limited to three patients and therefore may not be generalizable to broader populations. Individual variability in physiology, psychology, and treatment responses may have influenced the observed outcomes. Additionally, the lack of control or comparison group limits causal interpretation from the present work. Furthermore, anhedonia was assessed through clinical history taking rather than through a standardized instrument.

## 5 Conclusion

Given that reduction in tirzepatide dose alleviated anhedonia symptoms in each case in this study, there is increased confidence both that a) GLP1RA-treatment is driving this effect, and b) this effect is mediated by treatment dose, although this study is not designed or powered to ascribe any determinative conclusions. Clinicians can consider titrating patient doses based on reported symptom feedback with or without accessory pharmacological treatments that have demonstrated efficacy in reducing anhedonia-like symptoms (i.e. bupropion). With the pending approval of additional dual and triple agonists in the coming years that results in metabolic effects as or more profound than tirzepatide [51], awareness of the neurocognitive mechanisms by which the GLP1RA class influence reward-perception will remain an important part of comprehensive care in this patient population.

### 5.1 Key clinical messages

1. Anhedonia-like symptoms, including reduced motivation, emotional “flatness,” and loss of interest in previously enjoyable activities, may occur in some patients receiving high-dose tirzepatide.
2. Clinicians should consider routine assessment of changes in motivation, reward perception, and engagement in daily activities during GLP-1RA treatment, particularly at higher maintenance doses and after prolonged therapy.
3. Dose reduction of tirzepatide, with or without adjunctive therapy such as bupropion, may improve anhedonia-like symptoms while maintaining weight-loss benefits.

## 6 Patient perspective

### 6.1 Patient 1

Patient 1 declined to provide a patient perspective.

### 6.2 Patient 2

“After being on a GLP-1 medication for an extended period, I started to feel what I can only describe as “the mehs.” While I initially had success with weight loss, over time I noticed a growing sense of fatigue and a lack of interest in many of the activities that normally help my life function—things like working out, staying active, and even day-to-day responsibilities. This loss of motivation seemed to stall my progress, and my weight loss plateaued. I felt like I was moving through life on an assembly line but not enjoying any of the things I had worked so hard for (like increased mobility and active time with my children). After discussing these changes with my doctor, we adjusted my treatment plan by lowering my GLP-1 dose and adding [bupropion]. The difference was significant and immediate. I began to feel more like myself again—my energy improved, my interest in exercise and daily activities returned, and I was able to resume losing weight. This adjustment helped me find a better balance between managing my health and maintaining my overall quality of life.”

### 6.3 Patient 3

“I started taking [tirzepatide] in the fall of 2023 and moved up to highest dose fairly quickly. I had no side effects and have lost just under 190ish pounds. I did notice when I was filling out forms and was asked about depression, I always hesitated a bit in that. I can't say I was depressed, but I did notice that I kind of felt flat. I had stopped doing major food prep as I had been doing for the last two years and just ate properly but very basic. Same with exercise just doing what I considered to be the minimum. In December 2025. I went off [tirzepatide] for 4 weeks because I had skin removal surgery and I started back on a lower dose. I noticed that my mood changed. I was back to enjoying my food prep and looking forward to exercising again."

## Disclosures

SN and SK are full-time employees of Vineyard Health. SN has served as advisor for Novo Nordisk. GMT is a part-time employee of Vineyard Health.

## CRediT author statement

SN and SK conceptualized the submission. SN, ZAK, and GMT wrote the manuscript. SN, SK, ZAK, and GMT reviewed, edited, and approved the final submission and publication.

## Ethics review

Written informed consent was obtained from all patients for publication of this case report. Ethical approval was not required.

## Declaration of Artificial Intelligence (AI)

An AI tool (ChatGPT) was used to generate the graphical abstract based on the manuscript content. The authors reviewed all content and take responsibility for its content.

## Source of funding

No funding was received for this project.
